# Ensuring Equitable Application of Interventions to Vulnerable Subpopulations in the Kentucky Consortium for Accountable Health Communities (KC-AHC)

**DOI:** 10.13023/jah.0601.04

**Published:** 2024-09-01

**Authors:** Jing Li, Jessica M. Clouser, Akosua Adu, Aiko Weverka, Nikita Vundi, Terry D. Stratton, Mark V. Williams

**Affiliations:** Washington University in St Louis; University of Kentucky; University of Kentucky; University of Kentucky

**Keywords:** Appalachia, accountable health communities, AHCs, health equity, health disparities, health-related social needs, social determinants of health, SDOH

## Abstract

**Introduction:**

The Centers for Medicare and Medicaid Services (CMS) has funded the Accountable Health Communities (AHC) model to test whether systematically identifying and addressing the health-related social needs (HRSNs) of individuals would impact healthcare utilization and total cost of care for Medicare and Medicaid beneficiaries. Toward this effort, AHCs implement screening, referral, and community navigation services in their local areas. There are 28 CMS-funded AHCs nationwide, including the Kentucky Consortium for Accountable Health Communities (KC-AHC).

**Purpsoe:**

This study aims to assess the equity of KC-AHC model activities in three vulnerable subpopulations: dual enrollees, disabled individuals, and women.

**Methods:**

Twenty-eight primary care clinical sites across 19 healthcare organizations administered (inperson or telephonic) the AHC screening instrument from August 2018 to April 2021. Every six months, social needs positivity rates, navigation eligibility, service opted-in rates and delivery data were monitored among dual enrollees, disabled persons, and women. Subpopulations were compared to their comparisons (for example, non-dual enrollees) and to available benchmarked data.

**Results:**

All proportions of subpopulation in screened beneficiaries approximated or exceeded regional benchmarks. While needs among groups fluctuated over time, most reflected positivity rates in excess of comparisons: (1) rates among females ranged from 29.6% to 36.1%, but tended to narrow (relative to males) over time; (2) disabled individuals’ positivity rate ranged from 27.8% to 36.1% but also lessened over time compared with non-disabled counterparts; and (3) positive rates among the dually-enrolled ranged from 34.7% to 42.4%, with the disparity to non-dual enrollees remaining relatively stable. Rates of opt-in and receipt of navigation in dual enrollees and women did not show disparities. There was a persistent gap in opt-in rates between disabled and non-disabled beneficiaries, though one was not identified in receipt.

**Implications:**

Results suggest that the KC-AHC adequately screened dual enrollees, disabled individuals, and women during model implementation. The AHC Model may have helped to narrow gaps in social needs between sub-populations and comparison groups, with beneficiaries becoming better connected to community services.

## INTRODUCTION

Social factors drive pervasive health inequalities, contributing to elevated disease burden and healthcare utilization among vulnerable populations.^1^–^3^ Health-related social needs (HRSNs)—such as food insecurity, housing instability, transportation difficulties, lack of consistent utilities, and interpersonal violence—are associated with lower rates of receiving preventive care,^4^ lower rates of adherence with prescribed medications,^5^ and higher rates of chronic conditions.^6^ Referral to community services has been shown to reduce levels of HRSNs, as well as influence overall health and healthcare spending. Studies document significant reduction in HRSNs and improvement in patient health among those who receive navigation services to connect them with resources to meet their social needs.^7^–^10^ The Centers for Medicare and Medicaid Services (CMS) has funded the Accountable Health Communities (AHC) model to test whether systematically identifying and addressing HRSNs impacts on healthcare utilization and total cost of care for Medicare and Medicaid beneficiaries. Key components of the AHC model include identifying individuals’ HRSNs through universal screening conducted in clinical settings (e.g., at a primary care clinic or emergency department); connecting patients with community resources and services that can address their identified social needs; and assisting individuals in navigating these services, if indicated. Twenty-eight organizations nationwide were funded to implement screening,^11^ referral, and community navigation services. Notably, the AHC model was implemented in various communities across the U.S., with outcomes and effectiveness potentially differing based on local circumstances and resources. The Kentucky Consortium for Accountable Health Communities (KC-AHC) applied an academic–practice–community collaborative approach to screening and navigating CMS beneficiaries for HRSNs in a 29-county geographic target area (GTA) within Appalachian Kentucky.^12^ This area is largely rural—Census tracts in the study region were most frequently classified as small towns (34.5%) or rural areas (32.7%), with 28.7% and 4.1% classified as micropolitan and metropolitan, respectively.^13^ Twenty-five out of 29 of the counties are classified as having persistent poverty.^14^ To ensure that the model was administered equitably and did not inadvertently contribute to health and social disparities, the KC-AHC identified three specific subpopulations at risk of health disparities: dual Medicare and Medicaid enrollees,^15^,^16^ disabled individuals,^17^–^20^ and women.^21^,^22^ Multiple criteria were used to identify the populations of interest, including examination of existing data sources related to community needs and disparities; assessment of adequate subpopulation sizes to support the model’s screening and intervention goals; and the recommendations of the model’s consortium partners based on their local knowledge of their respective catchment areas.

The analysis aimed to (1) continuously assess whether model activities (e.g., screening and navigation) were equitably applied to these subpopulations; (2) initiate needed strategies to engage and retain adequate numbers of these subpopulations; and (3) assess whether these populations experienced HRSN disparities compared to their counterparts.

## METHODS

Project implementation years were categorized as Y2 (8/1/2018 – 4/30/2019), Y3 (5/1/2019 – 4/30/2020), and Y4 (5/1/2020 – 3/31/2021). Due to extended project planning, Year 2 encompassed a nine-month period beginning in August. Similarly, Year 4 concluded in March, comprising 11 months of data collection, in alignment with the project reporting timeline. Beneficiaries could be screened in multiple project years. This study was approved by the University’s Institutional Review Board (protocol #44566).

### HRSN Screening and Navigation Procedures

From August 2018, twenty-eight primary care clinical sites across 19 health care organizations administered (in person or telephonically) the AHC screening instrument following its standard procedures.^11^ Among these clinical sites, 11 are either federally qualified health centers or rural health clinics, while the remaining sites are affiliated with healthcare systems. This screening tool was developed by the CMS and was used by all AHC awardees. The core questions assess presence of food, housing, transportation, utilities, and personal safety needs, along with capturing demographic information (sex, age, household income, and race/ethnicity) (See Supplemental File 1 [SF1] in Additional Files online). If the beneficiary indicated any needs and self-reported two or more emergency department visits in the past 12 months (i.e., high risk), the navigation service was offered, which included one personal interview, a tailored action plan, and follow-ups. If a beneficiary opted in, the navigation was initiated to assist with accessing community services.

### Population Studied

The AHC model aimed to conduct universal HRSN screening to all community-dwelling CMS beneficiaries living in the GTA. The population in this study consisted of community-dwelling Medicare and/or Medicaid beneficiaries who sought medical care, either in person or virtually, at one of the KC-AHC primary care clinical sites. Only respondents who were aged 18 year or older at the time of the survey and responded to one or more HRSN screening question were retained.

### Subgroup Definitions

Three vulnerable subpopulations—dual enrollees, disabled individuals, and women—were identified to assess the equitability of HRSN screening and navigation activities:

- *Dual Enrollees*. Enrolled in both Medicare and Medicaid. Dual enrollment status was based on insurance information in the participating clinics’ patient profiles.
- *Disability Status*. Younger than 65 years with Medicare. Disability status can be defined differently depending on the context (e.g., purpose, setting, population), leading to variations in reporting. Because disability status was not an explicit component of the AHC screening tool, nor is it routinely collected among beneficiaries at clinical sites, a proxy measure was used to evaluate progress with this subpopulation. Specifically, those younger than aged 65 with Medicare were classified as disabled, as this is a legal classification indicating individuals meet the Social Security Administration’s criteria for disability. This serves as replicable measure for younger people with long-term disabilities, although it misses those with temporary disabilities and those aged 65 years or older with disabilities.
- *Gender*. Self-identify as female most frequently in HRSN screening (response option: male v. female). While gender is often a complex and diverse aspect of one’s identify, the AHC screening tool used a binary measure reflecting biological sex. Because gender can be fluid, we used measures of gender based on the most frequently reported gender in the screenings. Beneficiaries who never reported gender (4.6%) were removed from this subset of the analysis.

### Data and Analysis

The screening and navigation data used were derived from the monthly data report generated from the AHC Data System, a web-based case management application specifically designed for the AHC Model by the CMS to document patient screening and navigation activities. As expected, the three subpopulations are more prevalent in the 29-county GTA compared to national and statewide estimates using publicly available data sources (See Supplemental File 2 [SF2] in Additional Files online). Estimated benchmark values for the 29 KC-AHC counties (weighted averages) were used to evaluate equitable subpopulation representation in KC-AHC screening and navigation activities. To the extent that HRSN screening numbers/percentages for each subpopulation aligned with existing data on expected prevalence of these groups (SF2), engagement (i.e., participation in HRSNs screening) was considered sufficient relative to benchmark values. Every six months, the research team compared whether or not: (1) representation of subpopulations in screening activities reflected their proportions in the population-at-large within the GTA; (2) subpopulations had higher rates of core HRSNs relative to their comparison groups (i.e., not belonging to the subpopulation); (3) disparities in HRSN rates between subpopulations and comparison groups changed over time; (4) subpopulations who met navigation eligibility were equally represented; (5) subpopulations had lower rates of opt-in to navigation services; (6) subpopulations who opted in to navigation services received fewer services.

Descriptive statistics (frequencies and proportions) were used to describe prevalence of screening activities and outcomes, navigation case opt-in rates, and encounter activities for each subgroup across project years. A chi-squared test was used to examine HRSN disparities between subgroups and their counterparties in each project year. Observed changes in disparities on HRSN over project years were used to monitor potential trends.

### Strategies to Increase Equity

In preparation for implementation, strategies to ensure equity of model implementation activities included regularly reviewing publicly available benchmark data and amending the screened and navigated subpopulation target numbers as needed; compiling data to inform progress on screening and navigation activities for subpopulation beneficiaries; and utilizing staff at clinical sites with specialized training in cultural competence and/or prior professional experience with the targeted subpopulations.

As the project and its performance monitoring activities progressed, the KC-AHC team and stakeholders implemented additional change strategies and population-specific outreach efforts when certain subpopulations were found to be underrepresented and/or disparities were found in HRSN or navigation rates between subpopulations and their comparison groups. Such strategies included prioritizing dual enrollees for screening; highlighting housing resources on a community-resource inventory website; and employing a targeted effort to identify home assistance services and/or adult day centers for disabled residents. The study team also exercised an ongoing process of refining and optimizing the uptake and delivery of navigation services. These efforts encompassed the development of patient engagement materials, such as clinic posters, the dissemination of successful story videos to raise community awareness and engagement, and the recruitment of volunteers to deliver the initial navigation component before patients left the clinic.

## RESULTS

A total of 32,055 unique Medicare and Medicaid beneficiaries encompassing the three subpopulations were screened across all project years: 2,812 dual enrollees (8.8%); 5,575 beneficiaries younger than 65 with Medicare (17.4%); and 20,284 women (63.3%). The study population had a median age of 50.8 years, and the majority were non-Hispanic (92%), white (94%), and had an annual household income less than $15,000 (59%).

### Dual Enrollees

Approximately 8.6%, 9.8%, and 12.1% of beneficiaries screened at least once in Y2, Y3, and Y4, respectively, were dual enrollees (Table 1). This is less than the 13.1% benchmark for the 29-county KC-AHC study (SF2), however movement was in the right direction and may suggest that mitigation efforts (i.e., strategies to increase equity) narrowed this disparity. Dual enrollees had higher rates of core HRSNs (42.4% v. 32.1% in Y2, *p* < .0001; 34.7% v. 25.2% in Y3, *p* < .0001; 38.2% v. 29.9% in Y4, *p* = .0028), as well as rates of being high-risk status (22.1% v. 16.7% in Y2, *p* < .0001; 22.7% v. 16% in Y3, *p* < .0001; 48.1% v. 36% in Y4, *p* < .0001) than their non-dual enrollee counterparts (Table 1). Across all groups, food consistently emerged as the most frequently reported need, while safety was least commonly mentioned. A decline in reported needs was observed between Y2 and Y3, followed by an increase in Y4. This fluctuation could potentially be attributed to either heightened vulnerability associated with the COVID-19 pandemic or expanded sampling of patients whose clinical encounters occurred in emergency departments. Between Y2 and Y3, the data also showed a relative decline in disparities between groups for every core HRSN, except housing. This suggests that the general gap between dual enrollees and non-dual enrollees may be lower in Y3 than in Y2 (ranging from 0.8% to 3.8% lower), but that inequities in access to housing are potentially more persistent. Therefore, housing resources were emphasized in the community resource inventory and more effort targeted navigation activities to eligible dual enrollee beneficiaries. In Y4, the gap appeared to have narrowed for food and housing needs but widened for utilities and transportation. It is possible that downstream effects from the pandemic contributed to these gaps; for example, with social distancing guidelines in place, ridesharing among family or friends may have been avoided, thus removing a source of transportation for many beneficiaries. Similarly, the furloughs and job losses seen during the pandemic would likely hit utilities as an early indicator of financial hardship.

The navigation data (Table 4) showed that dual enrollees were approximately or adequately represented (11.9% in Y2, 14.8% in Y3, and 15% in Y4) relative to their benchmark (13.1%; see SF2)—suggesting the focus on recruiting dual enrollees into the screening process was an effective strategy. Overall rates of opt-in and subsequent receipt of navigation services were consistent among groups, further suggesting lack of disparities.

### Disability Status

For the KC-AHC region, an estimated 11.9% of Medicaid and Medicare beneficiaries were younger than 65 years with Medicare (SF2). This benchmark was exceeded, with 16.8%, 17.4%, and 15.3% of beneficiaries screened at least once in Y2, Y3, and Y4, respectively (Table 2). Higher rates of any HRSNs in this disability group were also observed for Y2. However, this disparity appeared to have narrowed over time (36.1% v. 32.4% in Y2, *p* = .0047; 27.8% v. 25.7% in Y3, *p* = .1206; 29.5% v. 31.2% in Y4, *p* = .493). In Y3, except for food, rates of positive HRSNs were identical or slightly lower in the disability group compared to the comparator group. This trend continued in Y4; the subpopulation appeared to have a lower rate of need than the comparison group for food (17.6% v. 18.7% *p* = .5777), housing (13.3% v. 13.8% *p* = .7773) and transportation (8.8% v. 9.3%, *p* = .7642).

As shown in Table 4, individuals younger than 65 with Medicare were appropriately represented, accounting for 18.5% (Y2), 17.5% (Y3), and 14.2% (Y4) of all navigation cases and aligning with their benchmark of 11.9% (SF1). We observed differences in the opt-in rate for those younger than 65 with Medicare and the comparison group. In Y2 the gap in opt-in rates was 4.5% (41.6% for those younger than 65 with Medicare vs. 46.1% for all others), in Y3 the gap in opt-in rates was 5.6% (66.7% for those younger than 65 with Medicare vs. 72.3 % for all others), but in Y4 this gap shrunk to 2% (85.7% for those younger than 65 with Medicare vs. 87.7% for all others).

### Gender

Women were well represented in the screenings, with over 60% of beneficiaries reporting their gender as female across all three project years (Table 3), surpassing the 51.1% benchmark indicated in Supplemental File 2. Women consistently reported having any HRSN at higher rates than men (36.1% v. 32.6% in Y2, *p* = .0018; 29.6% v. 25.9% in Y3, *p* = 0.0011; 31.6% v. 30.6% in Y4, *p* = 0.5967) (Table 3). The data also revealed that women were more likely to meet the classification of high risk relative to men in Y2 (18.8% v. 15.7%, *p* < .0001) and Y3 (18.5% v. 15.8%, *p* = .0016), but interestingly not in Y4 (38.3% v. 36.8%, *p* = .4839). The Y3–Y2 difference column in Table 3 suggests that gender disparity in need rates did not narrow over time and, if anything, increased slightly for several metrics. The Y4–Y3 difference column, however, suggests that the gap narrowed and was virtually erased for some HRSNs.

As shown in Table 4, women accounted for more than 60% of navigation cases in all three project years, outperforming their regional benchmark (51.1%; see SF2). Rates of opt-in to navigation services between females and males differed only slightly (42.3% v. 46.6% in Y2; 73.0% v. 70.4% in Y3; 86.6% v. 87.9% in Y4) among groups and were not consistently higher or lower for women across the project years. Additionally, receipt of navigation services for those opting in appear to be consistent among groups, suggesting a lack of disparities in receiving service offers from navigators.

### Navigation Trend

Throughout the study, there was a notable and continuous improvement in the uptake of navigation services (Y2 = 45.3%; Y3 = 71.3%; Y4 = 87.4%). Consistent and incremental enhancements were also observed in the delivery of navigation activities (any activity: Y2 = 75.0%, Y3 = 89.7%, Y4 = 99.3%; meaningful activity: Y2 = 62.3%, Y3 = 86.9%, Y4 = 98.8%). These improvements signified a positive trend in fostering a sustained increase in the use of navigation services and a trend toward KC-AHC objectives and goals.

## DISCUSSION

Results suggest that the KC-AHC adequately screened dual enrollees, disabled individuals, and women during the model implementation. Further, the analysis showed consistent disparities between these subpopulations and the comparison groups for most HRSNs—reflecting the heightened vulnerability of these subpopulations to social and community-level factors. For some groups (dual enrollees and disabled), a possible narrowing of the disparity was observed among the subpopulation and comparison over time, movement in the favorable direction. Similarly, although disparities between women and men grew between Y2 and Y3, they narrowed in Y4. This suggested that the potential benefits of project activities were equitably distributed and may have reduced disparities in social needs across target subpopulations.

The results of analysis of navigation activities suggest few differences in opt-in rates between subpopulations and counterparts, with the possible exception of those aged younger than 65 years with Medicare, the proxy for long-term disability. This persistent gap in opt-in rates between disabled and non-disabled beneficiaries is concerning in that it suggests that those in the subpopulation group may feel less comfortable opting in or that there are other barriers to participation in navigation services. However, the fact that this opt-in gap narrowed in Y4 suggests that targeted, population-specific outreach efforts to this subpopulation may have had the desired effect. Alternatively, the shifts in model implementation that occurred during Y4 (e.g., telephonic screening and navigation efforts, recruitment after an emergency department visit) or the increased strain of pandemic may have stimulated interest in opting into navigation services.

Only very minor differences were observed in the rate at which interested beneficiaries received navigation services between subpopulations and counterparts. This is encouraging in that it suggests that navigation services are being offered equitably, despite upstream barriers to ensuring equal access to initial screening services. The significant progress shown in the implementation of navigation services underscores the critical importance of continuous learning and adaptation in response to new data and experiences.

Y4 data suggest the absolute differences in HRSN rates between comparison groups and subpopulations are much smaller than observed in previous years. This is likely attributable to a combination of factors. One reason could be that the AHC Model is having the intended impact, with beneficiaries becoming better connected to community services; thus a reduction in their social needs is seen.

However, the impacts of COVID-19—both the widespread increase in financial need as well as the extension of social safety infrastructure—may have contributed to this effect. If so, such public health emergencies further underscore the need to systematically identify and address HRSNs to reduce health disparities and achieve health equity.

To accomplish this aim, systems approaches^23^—designed to shift interconnected aspects of public health problems—are needed. Indeed, the AHC model requires cross-sector partnerships that aim to improve health outcomes for vulnerable populations by addressing HRSNs to reduce health inequities. Community engagement and partnerships,^24^ have also been critical parts of the KC-AHC project, with community stakeholders being instrumental in helping to interpret subpopulation inclusion performance, identify root causes for observed disparities, and develop population-specific change strategies to ensure equity. As a result, the desire to reduce disparities and enhance equity shaped the KC-AHC implementation in several important ways. For example, staffing choices for screeners and navigators were influenced by the drive to increase equity, clinical sites recruitment to ensure access to targeted subpopulations, and capacity-enhancing efforts were based on and acted upon within community-specific assessments of local assets and needs.

### Limitations

As with any research, there are limitations to this study. First, there are large gaps in existing data related to the HRSNs of the targeted subpopulations relative to other community-dwelling Medicaid and Medicare beneficiaries. Although it is known that dual enrollees, disabled individuals, and women suffer health disparities generally, existing data on these types of inequalities within the beneficiary population are lacking. Therefore, it cannot be determined whether the disparities observed in study Year 2 should be considered as benchmarks or reference points. Second, despite quality-control activities to ensure the accurate compilation and use of secondary insurance information provided by clinical site partners, incomplete insurance information may still have been a potential source of error, particularly for the dual enrollees. Therefore, the data on subgroups are only as accurate as available insurance information. Third, while the focus on selected target subpopulations (dual enrollees, disabled individuals, and women) was guided by knowledge of state-wide health disparities and health issues of special concern in Kentucky, inherent characteristics of the study population precluded comparisons of race and ethnicity. Fourth, the standardized screening tool offered limited and somewhat simplistic response options for gender, and it lacked a self-reported disability status. Fifth, although the tracking of rate and disparities across subpopulations over time is a project strength, the effects of the COVID-19 pandemic not only created major shifts in AHC model implementation (i.e., shift to telephonic screening), but also in the vulnerabilities and needs of many beneficiaries. As a further complication, the resulting pandemic occurred during a focused effort shift to recruit patients from emergency departments. That these major changes occurred within the same quarter makes it difficult to untangle any limiting effects. Lastly, causal inferences should be made cautiously, as there may be unmeasured confounding factors at play and potential changes in clinical care related to the pandemic that have not been accounted for.

## IMPLICATIONS

Purposefully integrating equity-related activities with the AHC model implementation provided an opportunity to fill the data and knowledge gap and guide community alignment efforts. Such integration could also help ensure that health equity becomes a continuous pursuit—not merely a temporary, one-time focus. The integration of person-centered navigation services and community-level partner alignment strategies holds potential to provide equitable access and support to vulnerable populations and to help address HRSN disparities. Including community stakeholders in the conceptualization, development, and implementation is critical to attain meaningful improvements in health equity.

**SUMMARY BOX**

**What is already known about this topic?**

Referral to community services has been shown to reduce levels of health-related social needs (HRSNs), as well as influence overall health and healthcare spending. There is the concern that interventions are often unevenly applied, thereby exacerbating existing inequities.

**What is added by this report?**

The KC-AHC adequately screened dual enrollees, disabled individuals, and women during the model implementation. Purposefully integrating equity-related activities with the model implementation may have an impact on narrowed disparities in HRSN rates, with beneficiaries becoming better connected to community services.

**What are the implications for future research?**

Integration of person-centered navigation and community-level partner alignment strategies holds potential to provide equitable access and support to vulnerable populations and support address HRSN disparities.

## Supplementary Information





## Figures and Tables

**Table 1:** Summary of Dual Enrollee Prevalence and Screening Outcomes Compared to Non-dual Enrollees in Y2 (8/1/2018 – 4/30/2019), Y3 (5/1/2019 – 4/30/2020), and Y4 (5/1/2020 – 3/31/2021)

	Y2 (n = 18,473)			Y3 (n = 13,886)			Y4 (n = 5,410)				
Metric	Non-Dual Enrollee	Dual Enrollee	Y2 Diff	Non-Dual Enrollee	Dual Enrollee	Y3 Diff	Non-Dual Enrollee	Dual Enrollee	Y4 Diff	Y3 Diff - Y2 Diff	Y4 Diff - Y3 Diff
% (#) of total beneficiaries	91.4 (16,877)	8.6 (1,596)		90.2 (12,522)	9.8 (1,364)		87.9 (4,755)	12.1 (655)			

% (#) with any HRSN	32.1 (5,416)	42.4 (677)	−10.3*	25.2 (3,151)	34.7 (473)	−9.5*	29.9 (1,424)	38.2 (250)	−8.3*	0.8	1.2

% (#) with food need	22.8 (3,840)	31.5 (502)	−8.7*	17.9 (2,242)	23.6 (322)	−5.7*	18.2 (866)	20.9 (137)	−2.7	3	3

% (#) with housing need	13.7 (2,311)	16.7 (266)	−3*	9.7 (1,213)	13.3 (181)	−3.6*	13.4 (638)	15.9 (104)	−2.5	−0.6	1.1

% (#) with utilities need	9.1 (1,539)	11.5 (183)	−2.4*	6.1 (765)	7.3 (100)	−1.2	11.8 (559)	14.4 (94)	−2.6	1.2	−1.4

% (#) with transportation need	11.5 (1,935)	14.9 (238)	−3.4*	8.9 (1,113)	11.3 (154)	−2.4*	8.8 (419)	11.9 (78)	−3.1*	1	−0.7

% (#) with safety need	1.5 (246)	2.3 (36)	−0.8*	0.9 (118)	1.2 (16)	−0.3	0.4 (19)	0.6 (4)	−0.2	0.5	0.1

% (#) high risk†	16.7 (2,818)	22.1 (353)	−5.4*	16 (2,006)	22.7 (309)	−6.7*	36 (1710)	48.1 (315)	−12.1*	−1.3	−5.4

NOTES:

* Significant at *p* < .05.

† High risk is defined as having two or more emergency department visits in the past 12 months.

**Table 2:** Summary of Prevalence and Screening Outcomes and for Beneficiaries under Age 65 with Medicare Coverage in Y2 (8/1/2018 – 4/30/2019) and Y3 (5/1/2019 – 4/30/2020) and Y4 (5/1/2020 – 3/31/2021)

	Y2 (n = 18,473)			Y3 (n = 13,886)			Y4 (n = 5,410)				
Metric	Not Under Age 65 with Medicare	Under Age 65 with Medicare	Y2 Diff	Not Under Age 65 with Medicare	Under Age 65 with Medicare	Y3 Diff	Not Under Age 65 with Medicare	Under Age 65 with Medicare	Y4 Diff	Y3 Diff - Y2 Diff	Y4 Diff - Y3 Diff
% (#) of total beneficiaries	83.2 (15,362)	16.8 (3,111)		82.6 (11,463)	17.4 (2,423)		84.7 (4,582)	15.3 (828)			

% (#) with any HRSN	32.4 (4,970)	36.1 (1,123)	−3.7*	25.7 (2,951)	27.8 (673)	−2.1	31.2 (1,430)	29.5 (244)	1.7	1.6	3.8

% (#) with food need	22.9 (3,521)	26.4 (821)	−3.5*	18.2 (2,090)	19.6 (474)	−1.4	18.7 (857)	17.6 (146)	1.1	2.1	2.5

% (#) with housing need	13.8 (2,113)	14.9 (464)	−1.1	10 (1,152)	10 (242)	0	13.8 (632)	13.3 (110)	0.5	1.1	0.5

% (#) with utilities need	9.1 (1,394)	10.5 (328)	−1.4*	6.3 (722)	5.9 (143)	0.4	12 (551)	12.3 (102)	−0.3	1.8	−0.7

% (#) with transportation need	11.6 (1,779)	12.7 (394)	−1.1	9.2 (1,052)	8.9 (215)	0.3	9.3 (424)	8.8 (73)	0.5	1.4	0.2

% (#) with safety need	1.5 (227)	1.8 (55)	−0.3	1 (110)	1 (24)	0	0.4 (18)	0.6 (5)	−0.2	0.3	−0.2

% (#) high risk†	17 (2,618)	17.8 (553)	−0.8	16.6 (1,906)	16.9 (409)	−0.3	38 (1,741)	34.3 (284)	3.7	0.5	4

NOTES:

* Significant at *p* < .05.

† High risk is defined as having two or more emergency department visits in the past 12 months.

**Table 3:** Summary of Female Beneficiaries’ Prevalence and Screening Outcomes Compared to Male Beneficiaries in Y2 (8/1/2018 – 4/30/2019), Y3 (5/1/2019 – 4/30/2020), and Y4 (5/1/2020 – 3/31/2021)

	Y2 (n = 16,603)			Y3 (n = 12,388)			Y4 (n = 5,253)				
Metric	Male	Female	Y2 Diff	Male	Female	Y3 Diff	Male	Female	Y4 Diff	Y3 Diff-Y2 Diff	Y4 Diff-Y3 Diff
% (#) of total beneficiaries	34.5 (5,727)	65.5 (10,876)		36.3 (4,500)	63.7 (7,888)		37.3 (1,957)	62.7 (3,296)			

% (#) with any HRSN	32.6 (1,868)	36.1 (3,928)	−3.5*	25.9 (1,164)	29.6 (2,331)	−3.7*	30.6 (598)	31.6 (1,041)	−1	−0.2	2.7

% (#) with food need	22.9 (1,310)	26.2 (2,849)	−3.3*	18.3 (824)	21.2 (1,671)	−2.9*	18.9 (370)	18.8 (619)	0.1	0.4	3

% (#) with housing need	13.5 (776)	15.3 (1,659)	−1.8*	9.6 (433)	11.6 (915)	−2*	13.6 (267)	13.9 (459)	−0.3	−0.2	1.7

% (#) with utilities need	9.7 (557)	10.2 (1,109)	−0.5	6 (271)	7.2 (571)	−1.2*	12.2 (238)	12.2 (401)	0	−0.7	1.2

% (#) with transportation need	11.5 (657)	13.1 (1,430)	−1.6*	8.7 (390)	10.4 (823)	−1.7*	8.2 (160)	9.9 (325)	−1.7	0	0

% (#) with safety need	1.1 (61)	2 (213)	−0.9*	0.4 (17)	1.5 (117)	−1.1*	0.3 (5)	0.5 (17)	−0.2	−0.2	0.9

% (#) high risk†	15.7 (902)	18.8 (2,047)	−3.1*	15.8 (713)	18.5 (1,462)	−2.7*	38.3 (750)	36.8 (1214)	1.5	0.4	4.2

NOTES:

* Significant at *p* < .05.

† High risk is defined as having two or more emergency department visits in the past 12 months.

**Table 4:** Navigation Case Opt-in Rates and Encounter Activities for Non-dual Enrollees/Dual Enrollees, under Age 65 with Medicare Status and Female/Non-female Beneficiaries in Y2 (8/1/2018 – 4/30/2019), Y3 (5/1/2019 – 4/30/2020), and Y4 (5/1/2020–3/31/2021)

	Non-Dual Enrollee			Dual Enrollee		
Metric	Y2	Y3	Y4	Y2	Y3	Y4
% (#) of all navigation cases	88.1 (1,395)	85.2 (891)	85 (795)	11.9 (189)	14.8 (155)	15 (140)

% (#) of navigation cases opted in	45.7 (637)	70.9 (632)	87.9 (699)	42.3 (80)	73.5 (114)	84.3 (118)

Of navigation cases opted in, % (#) with any navigation activity carried out by BO	75.7 (482)	88.8 (561)	99.1 (693)	70 (56)	94.7 (108)	100 (118)

Of navigation cases opted in, % (#) with a meaningful* navigation activity carried out by BO	61.9 (394)	85.6 (541)	98.6 (689)	66.2 (53)	93.9 (107)	100 (118)

	**Not Under Age 65 with Medicare**			**Under Age 65 with Medicare**		
**Metric**	**Y2**	**Y3**	**Y4**	**Y2**	**Y3**	**Y4**

% (#) of all navigation cases	81.5 (1,291)	82.5 (863)	85.8 (802)	18.5 (293)	17.5 (183)	14.2 (133)

% (#) of navigation cases opted in	46.1 (595)	72.3 (624)	87.7 (703)	41.6 (122)	66.7 (122)	85.7 (114)

Of navigation cases opted in, % (#) with any navigation activity carried out by BO	75.5 (449)	89.4 (558)	99.3 (698)	73 (89)	91 (111)	99.1 (113)

Of navigation cases opted in, % (#) with a meaningful* navigation activity carried out by BO	61.7 (367)	86.7 (541)	98.7 (694)	65.6 (80)	87.7 (107)	99.1 (113)

	**Not Female**			**Female**		
**Metric**	**Y2**	**Y3**	**Y4**	**Y2**	**Y3**	**Y4**

% (#) of all navigation cases	31.6 (501)	34 (356)	39.3 (367)	68.4 (1083)	66 (690)	60.7 (568)

% (#) of navigation cases opted in	42.3 (212)	73 (260)	86.6 (318)	46.6 (505)	70.4 (486)	87.9 (499)

Of navigation cases opted in, % (#) with any navigation activity carried out by BO	73.1 (155)	89.6 (233)	98.7 (314)	75.8 (383)	89.7 (436)	99.6 (497)

Of navigation cases opted in, % (#) with a meaningful* navigation activity carried out by BO	60.8 (129)	87.3 (227)	98.4 (313)	63 (318)	86.6 (421)	99 (494)

* *Meaningful* indicates the case had at least one personal interview conducted, action plan created, or follow-up occurred.
