# Supplementary material for: Ensuring Equitable Application of Interventions to Vulnerable Subpopulations in the Kentucky Consortium for Accountable Health Communities (KC-AHC)
Source: J Appalach Health. 2024 Sep 1;6(1-2):38–56. doi: 10.13023/jah.0601.04 (PMC11617025; doi:10.13023/jah.0601.04)
Supplement: Supplementary file 1 [file jah-6-1-2-38-Additionalfile1.pdf]

The Accountable Health Communities Health-Related Social Needs Screening Tool

What's the Accountable Health Communities (AHC) Health-Related Social Needs (HRSN) Screening Tool?

We at the Centers for Medicare & Medicaid Services (CMS) Center for Medicare and Medicaid Innovation (CMMI) made the Accountable Health Communities (AHC) Health-Related Social Needs (HRSN) Screening Tool to use in the AHC Model.[1] We're testing to see if systematically finding and dealing with the health-related social needs of Medicare and Medicaid beneficiaries has any effect on their total health care costs and makes their health outcomes better.

Why is the AHC HRSN Screening Tool important?

Growing evidence shows that if we deal with unmet HRSNs like homelessness, hunger, and exposure to violence, we can help undo their harm to health. Just like with clinical assessment tools, providers can use the results from the HRSN Screening Tool to inform patients' treatment plans and make referrals to community services.

What does the AHC HRSN Screening Tool mean for me?

Screening for HRSNs isn't standard clinical practice yet. We're making the AHC HRSN Screening Tool a standard screening across all the communities in the AHC Model. We're sharing the AHC HRSN Screening Tool for awareness.

What's in the AHC HRSN Screening Tool?

In a National Academy of Medicine discussion paper,[2] we shared the 10-item HRSN Screening Tool. The Tool can help providers find out patients' needs in these 5 core domains that community services can help with:

- Housing instability
- Food insecurity
- Transportation problems
- Utility help needs

---

[1] United States, U.S. Department of Health and Human Services, Centers for Medicare & Medicaid Services. (2017, September 05). Accountable Health Communities Model. https://innovation.cms.gov/initiatives/ahcm.

[2] Billioux, A., MD, DPhil, Verlander, K., MPH, Anthony, S., DrPH, & Alley, D., PhD. (2017). Standardized Screening for Health-Related Social Needs in Clinical Settings: The Accountable Health Communities Screening Tool. National Academy of Medicine Perspectives, 1-9. https://nam.edu/wp-content/uploads/2017/05/Standardized-Screening-for-Health-Related-Social-Needs-in-Clinical-Settings.pdf.

- Interpersonal safety

In the final version below, we made small revisions to the original 10 questions based on cognitive testing we did since we shared the first version. In the final version we also included questions in 8 supplemental domains that we haven't shared before:

- Financial strain
- Employment
- Family and community support
- Education
- Physical activity
- Substance use
- Mental health
- Disabilities

Who should use the AHC HRSN Screening Tool?

The questions in the AHC HRSN Screening Tool are meant to be used for individual respondents who answer the questions themselves. A parent or caregiver can answer for an individual, too, if that makes more sense. Clinicians and their staff can easily use this short tool as part of their busy clinical workflows with people of all different ages, backgrounds, and settings.

In the next 5 years, hundreds of participating clinical delivery sites across the 32 AHCs will screen over 7 million Medicare and Medicaid beneficiaries using the 10 core domain questions. The AHCs can also choose to add any of the supplemental domain questions into their standard screening processes.

Who made the AHC HRSN Screening Tool?

We made this tool with a panel of experts from around the country including:

- Tool developers
- Public health and clinical researchers
- Clinicians
- Population health and health systems executives
- Community-based organization leaders
- Federal partners

We got permission from the original authors of the questions to use, copy, modify, publish, and distribute the questions for the AHC Model and for our use only.

AHC HRSN Screening Tool Core Questions

If someone chooses the underlined answers, they might have an unmet health-related social need.

Living Situation

1. **What is your living situation today?**[3]
   - ☐ I have a steady place to live
   - ☐ I have a place to live today, but **I am worried** about losing it in the future
   - ☐ I do not have a steady place to live (I am temporarily staying with others, in a hotel, in a shelter, living outside on the street, on a beach, in a car, abandoned building, bus or train station, or in a park)

2. **Think about the place you live. Do you have problems with any of the following?**[4]
   CHOOSE ALL THAT APPLY
   - ☐ Pests such as bugs, ants, or mice
   - ☐ Mold
   - ☐ Lead paint or pipes
   - ☐ Lack of heat
   - ☐ Oven or stove not working
   - ☐ Smoke detectors missing or not working
   - ☐ Water leaks
   - ☐ None of the above

Food

Some people have made the following statements about their food situation. Please answer whether the statements were OFTEN, SOMETIMES, or NEVER true for you and your household in the last 12 months. [5]

3. **Within the past 12 months, you worried that your food would run out before you got money to buy more.**
   - ☐ Often true
   - ☐ Sometimes true
   - ☐ Never true

---

[3] National Association of Community Health Centers and partners, National Association of Community Health Centers, Association of Asian Pacific Community Health Organizations, Association OPC, Institute for Alternative Futures. (2017). PRAPARE. http://www.nachc.org/research-and-data/prapare/

[4] Nuruzzaman, N., Broadwin, M., Kourouma, K., & Olson, D. P. (2015). Making the Social Determinants of Health a Routine Part of Medical Care. Journal of Healthcare for the Poor and Underserved, 26(2), 321-327.

[5] Hager, E. R., Quigg, A. M., Black, M. M., Coleman, S. M., Heeren, T., Rose-Jacobs, R., Frank, D. A. (2010). Development and Validity of a 2-Item Screen to Identify Families at Risk for Food Insecurity. Pediatrics, 126(1), 26-32. doi:10.1542/peds.2009-3146

4. **Within the past 12 months, the food you bought just didn't last and you didn't have money to get more.**
   - ☐ Often true
   - ☐ Sometimes true
   - ☐ Never true

Transportation

5. **In the past 12 months, has lack of reliable transportation kept you from medical appointments, meetings, work or from getting things needed for daily living?[6]**
   - ☐ Yes
   - ☐ No

Utilities

6. **In the past 12 months has the electric, gas, oil, or water company threatened to shut off services in your home?[7]**
   - ☐ Yes
   - ☐ No
   - ☐ Already shut off

Safety

Because violence and abuse happens to a lot of people and affects their health we are asking the following questions. [8]

7. **How often does anyone, including family and friends, physically hurt you?**
   - ☐ Never (1)
   - ☐ Rarely (2)
   - ☐ Sometimes (3)
   - ☐ Fairly often (4)
   - ☐ Frequently (5)

---

[6] National Association of Community Health Centers and Partners, National Association of Community Health Centers, Association of Asian Pacific Community Health Organizations, Association OPC, Institute for Alternative Futures. (2017). PRAPARE. http://www.nachc.org/research-and-data/prapare/

[7] Cook, J. T., Frank, D. A., Casey, P. H., Rose-Jacobs, R., Black, M. M., Chilton, M., . . . Cutts, D. B. (2008). A Brief Indicator of Household Energy Security: Associations with Food Security, Child Health, and Child Development in US Infants and Toddlers. Pediatrics, 122(4), 867-875. doi:10.1542/peds.2008-0286

[8] Sherin, K. M., Sinacore, J. M., Li, X. Q., Zitter, R. E., & Shakil, A. (1998). HITS: a Short Domestic Violence Screening Tool for Use in a Family Practice Setting. Family Medicine, 30(7), 508-512

8. **How often does anyone, including family and friends, insult or talk down to you?**
- ☐ Never (1)
- ☐ Rarely (2)
- ☐ Sometimes (3)
- ☐ Fairly often (4)
- ☐ Frequently (5)

9. **How often does anyone, including family and friends, threaten you with harm?**
- ☐ Never (1)
- ☐ Rarely (2)
- ☐ Sometimes (3)
- ☐ Fairly often (4)
- ☐ Frequently (5)

10. **How often does anyone, including family and friends, scream or curse at you?**
- ☐ Never (1)
- ☐ Rarely (2)
- ☐ Sometimes (3)
- ☐ Fairly often (4)
- ☐ Frequently (5)

A score of 11 or more when the numerical values for answers to questions 7-10 are added shows that the person might not be safe.

AHC HRSN Screening Tool Supplemental Questions

Financial Strain

11. How hard is it for you to pay for the very basics like food, housing, medical care, and heating? Would you say it is:[9,]

- ☐ Very hard
- ☐ Somewhat hard
- ☐ Not hard at all

Employment

12. Do you want help finding or keeping work or a job?[10]

- ☐ Yes, help finding work
- ☐ Yes, help keeping work
- ☐ I do not need or want help

Family and Community Support

13. If for any reason you need help with day-to-day activities such as bathing, preparing meals, shopping, managing finances, etc., do you get the help you need?[11]

- ☐ I don't need any help
- ☐ I get all the help I need
- ☐ I could use a little more help
- ☐ I need a lot more help

14. How often do you feel lonely or isolated from those around you?[12]

- ☐ Never
- ☐ Rarely
- ☐ Sometimes
- ☐ Often
- ☐ Always

---

[9] Hall, M. H., Matthews, K. A., Kravitz, H. M., Gold, E. B., Buysse, D. J., Bromberger, J. T., . . . Sowers, M. (2009). Race and Financial Strain are Independent Correlates of Sleep in Midlife Women: The SWAN Sleep Study. Sleep, 32(1), 73-82. doi:10.5665/sleep/32.1.73

[10] Identifying and Recommending Screening Questions for the Accountable Health Communities Model (2016, July) Technical Expert Panel discussion conducted at the U.S. Department of Health and Human Services, Centers for Medicare & Medicaid Services, Baltimore, MD.

[11] Kaiser Permanente. (2012, June). Medicare Total Health Assessment Questionnaire. Retrieved from https://mydoctor.kaiserpermanente.org/ncal/Images/Medicare%20Total%20Health%20Assessment%20Questionnaire_tcm75-487922.pdf

[12] Northwestern University. (2017). PROMIS Item Bank v. 1.0 – Emotional Distress - Anger - Short Form 1

Education

15. Do you speak a language other than English at home?[13]

- ☐ Yes
- ☐ No

16. Do you want help with school or training? For example, starting or completing job training or getting a high school diploma, GED or equivalent.[14]

- ☐ Yes
- ☐ No

Physical Activity

17. In the last 30 days, other than the activities you did for work, on average, how many days per week did you engage in moderate exercise (like walking fast, running, jogging, dancing, swimming, biking, or other similar activities)?[15]

- ☐ 0
- ☐ 1
- ☐ 2
- ☐ 3
- ☐ 4
- ☐ 5
- ☐ 6
- ☐ 7

18. On average, how many minutes did you usually spend exercising at this level on one of those days?[16]

- ☐ 0
- ☐ 10
- ☐ 20
- ☐ 30
- ☐ 40
- ☐ 50
- ☐ 60

---

[13] United States, US Census Bureau. (2017). American Community Survey. Retrieved from https://www.census.gov/programs-surveys/acs/

[14] Identifying and Recommending Screening Questions for the Accountable Health Communities Model (2016, July) Technical Expert Panel discussion conducted at the U.S. Department of Health and Human Services, Centers for Medicare & Medicaid Services, Baltimore, MD.

[15] Coleman, K. J., Ngor, E., Reynolds, K., Quinn, V. P., Koebnick, C., Young, D. R., . . . Sallis, R. E. (2012). Initial Validation of an Exercise "Vital Sign" in Electronic Medical Records. Medicine and Science in Sport and Exercise, 44(11), 2071-2076. doi:10.1249/MSS.0b013e3182630ec1

[16] Ibid

☐ 90
☐ 120
☐ 150 or greater

Follow these 2 steps to decide if the person has a physical activity need:

1. Calculate [“number of days” selected] x [“number of minutes” selected] = [number of minutes of exercise per week]
2. Apply the right age threshold:
   - Under 6 years old: You can’t find the physical activity need for people under 6.
   - Age 6 to 17: Less than an average of 60 minutes a day shows an HRSN.
   - Age 18 or older: Less than 150 minutes a week shows an HRSN.

Substance Use

The next questions relate to your experience with alcohol, cigarettes, and other drugs. Some of the substances are prescribed by a doctor (like pain medications), but only count those if you have taken them for reasons or in doses other than prescribed. One question is about illicit or illegal drug use, but we only ask in order to identify community services that may be available to help you. [17]

19. How many times in the past 12 months have you had 5 or more drinks in a day (males) or 4 or more drinks in a day (females)? One drink is 12 ounces of beer, 5 ounces of wine, or 1.5 ounces of 80-proof spirits.

☐ Never
☐ Once or Twice
☐ Monthly
☐ Weekly
☐ Daily or Almost Daily

20. How many times in the past 12 months have you used tobacco products (like cigarettes, cigars, snuff, chew, electronic cigarettes)?

☐ Never
☐ Once or Twice
☐ Monthly
☐ Weekly
☐ Daily or Almost Daily

[17] United States, U.S. Department of Health and Human Services, National Institutes of Health. (n.d.). Helping Patients Who Drink Too Much: A Clinician's Guide (2005 ed., pp. 1-34).

21. **How many times in the past year have you used prescription drugs for non-medical reasons?**
   - ☐ Never
   - ☐ Once or Twice
   - ☐ Monthly
   - ☐ Weekly
   - ☐ Daily or Almost Daily

22. **How many times in the past year have you used illegal drugs?**
   - ☐ Never
   - ☐ Once or Twice
   - ☐ Monthly
   - ☐ Weekly
   - ☐ Daily or Almost Daily

Mental Health

23. **Over the past 2 weeks, how often have you been bothered by any of the following problems?[18]**

    a. Little interest or pleasure in doing things?
   - ☐ Not at all (0)
   - ☐ Several days (1)
   - ☐ More than half the days (2)
   - ☐ Nearly every day (3)

    b. Feeling down, depressed, or hopeless?
   - ☐ Not at all (0)
   - ☐ Several days (1)
   - ☐ More than half the days (2)
   - ☐ Nearly every day (3)

If you get 3 or more when you add the answers to questions 23a and 23b the person may have a mental health need.

[18] Kroenke, K., Spitzer, R. L., & Williams, J. B. (2003). The Patient Health Questionnaire-2: validity of a two-item depression screener. Medical Care, 41(11), 1284-1292.

24. Stress means a situation in which a person feels tense, restless, nervous, or anxious, or is unable to sleep at night because his or her mind is troubled all the time. Do you feel this kind of stress these days?[19]

- ☐ Not at all
- ☐ A little bit
- ☐ Somewhat
- ☐ Quite a bit
- ☐ Very much

Disabilities

25. Because of a physical, mental, or emotional condition, do you have serious difficulty concentrating, remembering, or making decisions?[20] **(5 years old or older)**

- ☐ Yes
- ☐ No

26. Because of a physical, mental, or emotional condition, do you have difficulty doing errands alone such as visiting a doctor's office or shopping?[21] **(15 years old or older)**

- ☐ Yes
- ☐ No

---

[19] Elo, A.L., Leppänen, A., & Jahkola, A. (2003). Validity of a Single-Item Measure of Stress Symptoms. Scandinavian Journal of Work, 29(6), 444-451.
[20] United States, U.S. Department of Health and Human Services, Office of the Assistant Secretary for Planning and Evaluation (n.d.). (2011). Implementation Guidance on Data Collection Standards for Race, Ethnicity, Sex, Primary Language, and Disability Status. Retrieved from https://aspe.hhs.gov/basic-report/hhs-implementation-guidance-data-collection-standards-race-ethnicity-sex-primary-language-and-disability-status
[21] Ibid.
