# Supplementary material for: Ensuring Equitable Application of Interventions to Vulnerable Subpopulations in the Kentucky Consortium for Accountable Health Communities (KC-AHC)
Source: J Appalach Health. 2024 Sep 1;6(1-2):38–56. doi: 10.13023/jah.0601.04 (PMC11617025; doi:10.13023/jah.0601.04)
Supplement: Supplementary file 2 [file jah-6-1-2-38-Additionalfile2.docx]

**SF2. Subpopulation Benchmarks**

| **Geographic Unit** | **% of those with Medicare /Medicaid coverage that are dual enrolled**^a^ | **% of those with Medicare/Medicaid coverage that are age 19-64 with Medicare^b^** | **% Female^c^** |
| --- | --- | --- | --- |
| United States | 10.2 | 6.9 | 51.1 |
| Kentucky | 9.6 | 9.6 | 51.2 |
| 29 KC-AHC Counties | 13.1 | 11.9 | 51.1 |

*Benchmark measure definitions:*

*^a^Calculated as total dual enrollees from* [*MMCO Statistical & Analytic Report Annual Release 12/2018 Snapshot*](https://www.cms.gov/Medicare-Medicaid-Coordination/Medicare-and-Medicaid-Coordination/Medicare-Medicaid-Coordination-Office/Analytics) *divided by Medicare coverage alone or in combination (S2704_C02_002E) plus Medicaid/means-tested public coverage alone or in combination (S2704_C02_006E) minus dual enrollees. Medicare and Medicaid coverage numbers were from the* [*2018 5-year American Community Survey Table S704*](https://data.census.gov/cedsci/table?q=medicare&g=0100000US_0400000US21,21.050000&tid=ACSST5Y2018.S2704&moe=false&tp=false&hidePreview=true)*.*

*^b^This is one proxy for disability because to eligible for Medicare before age 65 beneficiaries must be eligible for disability benefits for >24 months, or have health condition such as amyotrophic lateral sclerosis or end state renal disease, generally. Calculated as the sum of the non-institutionalized civilian population age 19-64 with Medicare coverage alone or in combination (S2704_C02_004E) divided by Medicare coverage alone or in combination (S2704_C02_002E) plus Medicaid/means-tested public coverage alone or in combination (S2704_C02_006E) minus dual enrollees. Medicare and Medicaid coverage numbers were from the* [*2018 5-year American Community Survey Table S704*](https://data.census.gov/cedsci/table?q=medicare&g=0100000US_0400000US21,21.050000&tid=ACSST5Y2018.S2704&moe=false&tp=false&hidePreview=true)*. Dual enrollees were from the* [*MMCO Statistical & Analytic Report Annual Release 12/2018 Snapshot*](https://www.cms.gov/Medicare-Medicaid-Coordination/Medicare-and-Medicaid-Coordination/Medicare-Medicaid-Coordination-Office/Analytics)*.*

*^c^Calculated as the female non-institutionalized civilian population (S1810_C01_003E) divided by the total non-institutionalized civilian population (S1810_C01_001E) from the* [*2018 5-year American Community Survey Table S1810*](https://data.census.gov/cedsci/table?q=disability&g=0100000US_0400000US21,21.050000&tid=ACSST5Y2018.S1810&hidePreview=true)
